# Helicity dependent diffraction by angular momentum transfer

**DOI:** 10.1038/s41598-019-48923-6

**Published:** 2019-08-28

**Authors:** S. Deepa, Bhargava Ram B.S., P. Senthilkumaran

**Affiliations:** Indian Institute of Technology Delhi, Department of Physics, New Delhi, 110016 India

**Keywords:** Optics and photonics, Transformation optics

## Abstract

In this article we show that diffraction segregates the polarization singularities according to their handedness. Polarization singularities are superpositions of left and right handed circular polarization vortex states. In the superposition, the component states possess different orbital angular momenta depending on the type of the singularity. A fork grating that can generate different orbital angular momentum (OAM) states in different diffraction orders is shown to segregate right and left handed polarization singularities. A V-point polarization singularity that corresponds to one combination of OAM states incident on the fork grating is found to diffract in such a way that the same OAM combination does not occur in all the nonzero diffraction orders. As a result, each of the diffraction orders will have different polarization singularities. This OAM transfer by the fork grating segregates the right and left handed polarization singularities thereby, making the diffraction helicity dependent.

## Introduction

**Figure 1:**
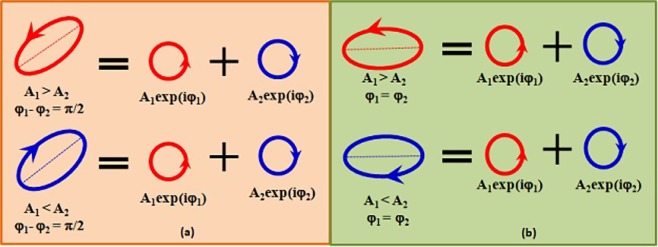
Right handed ellipse (row1) and left handed ellipse (row2) as superpositions of spin states. For eg. a right handed ellipse is actually a superposition of both right and left handed circular spin states where the handedness is dependent upon the amplitudes of the spin states as shown in conditions mentioned in (**a**) and (**b**). In this circular decomposition the component amplitudes decide the handedness and the component phases decide the azimuth(orientation) of the resultant SOP.

The paraxial diffraction phenomenon has never been known to be dependent on handedness. We report here for the first time that right handed and left handed polarization singularities diffract into different orders of a fork grating. This segregation according to handedness is due to the transfer of OAM. The phenomenon observed here is helicity dependent and not spin dependent. Helicity (handedness) is different from spin and it is defined as the sign of the projection of spin on the momentum vector of the beam. For example, a right elliptically polarized light has right handedness but can be decomposed into right and left handed circularly polarized (spin) states as shown in Fig. 1. Literature^1,2^ on the distinction between spin and helicity in optics are available. Spin dependent optics with meta-surfaces separating homogeneously polarized spin states based on nanostructured surfaces have been reported^3–7^, but what we report here is handedness dependent phenomenon. In fabricated nanostructures helicity locking through plasmonic vortex has been shown^8^. In the present work, the diffracting structures have sizes that are bigger by many magnitudes than the operating wavelength, where polarization is not expected to play any role. There are also no surface plasmons, no guided plasmonic modes, no nanostructured photonics involved. Here, both the incident and the diffracted fields through the fork grating possess polarization singularities.

We introduce some of the essential definitions and nomenclatures for the general audience before venturing into the main objective of this paper. A phase singularity, also called as a vortex, of charge ‘*m*’ carries an OAM of ‘*mℏ*’ per photon^9^. The charge ‘*m*’ of the phase singularity is defined by $\oint \nabla \varphi \cdot dl=m2\pi$, where *ϕ* denotes phase. A circularly polarized light carries Spin Angular Momentum (SAM) ‘*σℏ*’, where ‘*σ*’ defines the spin. A circularly polarized vortex beam has both SAM and OAM where SAM is arising due to circular polarization and OAM is arising due to helical ramp phase structure of the beam. A phase singularity has a helically varying phase distribution with circulating phase gradients (▿*ϕ*), whereas in a polarization singularity there is circularly varying azimuth gradient (▿*γ*).

**Figure 2:**
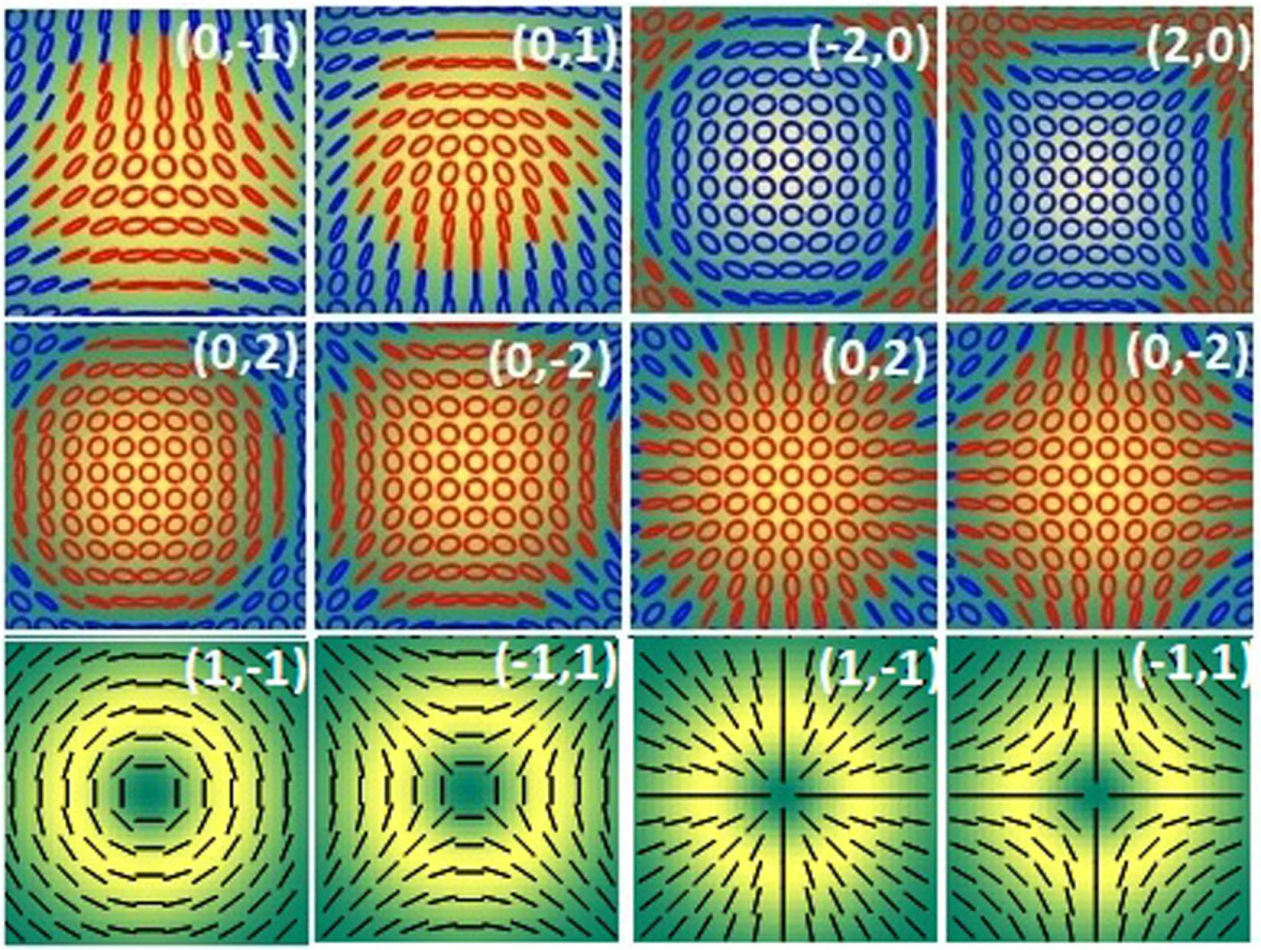
Row 1 and 2: SOP distributions of C-point singularities; Row 3: Four types of V-point singularities. (*m*_1_, *m*_2_) denotes the OAM states in right circular polarization (RCP) and left circular polarization LCP. Red and blue colors represent right and left handedness respectively. The background gives intensity.

Extensive studies on phase singularities have led way to a variety of polarization singularities in optical fields^9–12^. Polarization singularities namely C-points and V-points are the stationary points in spatially varying polarization distributions of ellipse and vector fields, respectively. At the singular point the azimuth (*γ*) is undefined. The integral, $\frac{1}{2\pi }\oint \nabla \gamma \cdot dl$ is non-zero for a polarization singularity, evaluated around the singularity. The Poincare-Hopf (PH) index (*η*) characterizing the vector fields and the *I*_*c*_ index characterizing the ellipse fields are defined by this integral. Undefined azimuth occurs where the Stokes parameters *S*_1_ = *S*_2_ = 0, which leads to two possibilities namely *S*_3_ = ±1 (circular polarization) or *S*_3_ = 0 (intensity null). If the state of polarization (SOP) at the polarization singular point is circular, then the singularity is a C-point. The V-points occur at intensity nulls. The generic ellipse field singularities are classified as lemons, monstars and stars, based on polarization distribution. The vector fields comprise of spatially varying linear polarization states, and the generic vector field singularities are classified into four types. Figure 2 shows some of the C-points and generic V-points. C-points can be further classified based on handedness *h*^±^. The handedness can be defined as the projection of spin on the propagation vector. Hence a C-point (lemon/star) with a given C-point index I_c_ can be right (*h*^+^) or left (*h*^−^) handed and can occupy any of the poles of the Poincare sphere^11,13,14^. The Poincare sphere, whose every point is used to represent the polarization states, represent linear polarization states by points along equator, circular polarization states by poles and elliptical polarization states by other points. The handedness of V-points is undefined as they are made of linear states. In what follows, we explain the connection between OAM and polarization singularity, the connection between OAM and handedness of the polarization singularity which leads to helicity dependent diffraction.

## Angular Momentum of Light and Polarization Singularity

The connection between phase and polarization singularity becomes clear by noting the homomorphism in the definition of charge of a phase singularity *m* and polarization singularity indices *I*_*c*_ and *η*. In circular basis representation of polarization states, the phase difference between orthogonal circular basis states decides the azimuth of the resulting polarization state. Hence by having appropriate phase difference gradient in the component states, the required ▿*γ* can be realized in the polarization distribution. In other words, these singularities (C-points and V-points) can be seen as superposition of two beams with different OAM in opposite SAM states^15,16^. In circular basis, a generic C-point singularity is represented as a superposition of OAM states *m* = 0 and *m* = ±1 in orthogonal SAM states. Here *m* is the topological charge of the phase singularity. Similarly a generic V-point is a superposition of OAM states with *m* = +1 and *m* = −1 in opposite SAM states. The net or total angular momentum (SAM + OAM) of a V-point singularity is zero whereas it is a non- zero quantity for C-points. If the topological charge of the superposing beams is such that, |*m*| ≥ 2 it will lead to generation of corresponding higher order singularities. The distinction between C and V-points when |*I*_*c*_| = |*η*|, can be made by a limiting process taking into consideration the neighbourhood SOP and the Stokes phase distributions^17,18^. The Stoke’s phase distribution is generally given by the argument of the Stokes field *S*_12_ constructed from the well known Stokes parameters. The fact that polarization singularity contains phase singularities is evident by circular decomposition. Hence by inserting a circular polarizer into a polarization singularity, the phase singularity can be seen. This is a scalar field singularity. In the inhomogeneous polarization distributions containing a closed L-line, the insertion of a polarizer will reveal the presence of phase singularity at a point on the L-line^19,20^.

## Helicity and OAM

The helicity of the polarization singularity is decided by the OAM combination. Consider a C-point which is a superposition of a Laguerre Gaussian (LG) beam with azimuthal index 0 (Gaussian beam) in right circular polarization (RCP) and LG beam with azimuthal index 1 in left circular polarization (LCP). In the superposition at the singular point, the LCP component is absent as the LG beam has vortex phase which demands the presence of intensity null at the phase singular point. Hence the handedness of the C-point is decided by the handedness of the circular polarization of the Gaussian beam which is right handed here. In the immediate neighborhood of a C-point, the phase difference between the left and right circular component states decides azimuth variation and the amplitude difference between them decides the ellipticity variation. This is a bright C-point.The superposition of two LG beams in orthogonal circular polarization states - one with charge *m* vortex (*m* ≠ 0) and the other with charge *m* ± 1 vortex, results in a dark C-point^21,22^. Therefore, a C-point can occur at any intensity level. For the formation of V-point, superposition of two vortex beams with charge +*m* and −*m* in left and right circular polarization components, leads to SOP distribution which is linear. Both the superposing beams have same amplitude variation and hence the ellipticity is zero all across the beam and the azimuth rotates about the V-point singularity due to phase difference between opposite charged vortices. Generally when one of the beam components is Gaussian, the resultant beam acquires the handedness of the Gaussian beam of lower order. When two vortex beams combine, the resultant always acquires the handedness of that beam which possesses lower OAM value. For the polarization singularities, Freund’s definition relating the charge of the vortex, *I*_*c*_ index and handedness of the singularity can also be used to decide the handedness of the polarization singularity^23^. The C points can be characterized by their photon handedness (*h*^±^). The C point index can also be given as, ${I}_{c}=-\frac{{h}_{R/L}{m}_{R/L}}{2}$, where *h*_*R*_ = +1 and *h*_*L*_ = −1. A right/left handed (*h*^+^/*h*^−^) C-point corresponds to a vortex of charge *m*_*R*/*L*_ in left/right (*h*_*L*_/*h*_*R*_) circular polarization^23^. For example, a lemon can be realized using either a positive or negative vortex. Using this formula, we can figure out that a positive vortex can produce a lemon only in the South pole and not in the North Pole. In other words, if *I*_*c*_ index is fixed, change in the polarity of the OAM state should be accompanied by change in the handedness^14^.

## Angular Momentum Transfer by Diffraction Through a Fork Grating

Diffraction optics is a scalar optics and is used in many optical experiments to create desired phase and intensity profiles. It is well established that in scalar optics, phase carries more information than amplitude^24^. Since phase and polarization are closely related to each other it is natural to expect polarization also to carry information like phase^25^. The spatial information appears in the form of spatially varying polarization distribution, making this field a potentially rewarding area of research. For example, even though a V-point singularity has a plane wavefront, the circular polarization basis decomposition shows that it is tantamount to be made of two opposite helical wavefronts^15,26^. When the vectorial nature of the beam is concerned, both phase and polarization can be coupled to modulate a light beam in a structured way.

In this paper we show that diffraction segregates the polarization singularities according to their handedness. By using a fork grating we show that a V-point diffracts into dark and bright C-points. OAM transfer makes the diffraction’ *helicity dependent*’. The Stokes index in the incident and diffracted beams are observed to be the same. This helicity dependent diffraction is different than the one in which V-point disintegrates into lower index C-points^27,28^. In both diffraction experiments, we observed that since the incident V-point is devoid of any handedness, the diffracted beams have equal number of right and left handed singularities.

**Figure 3:**
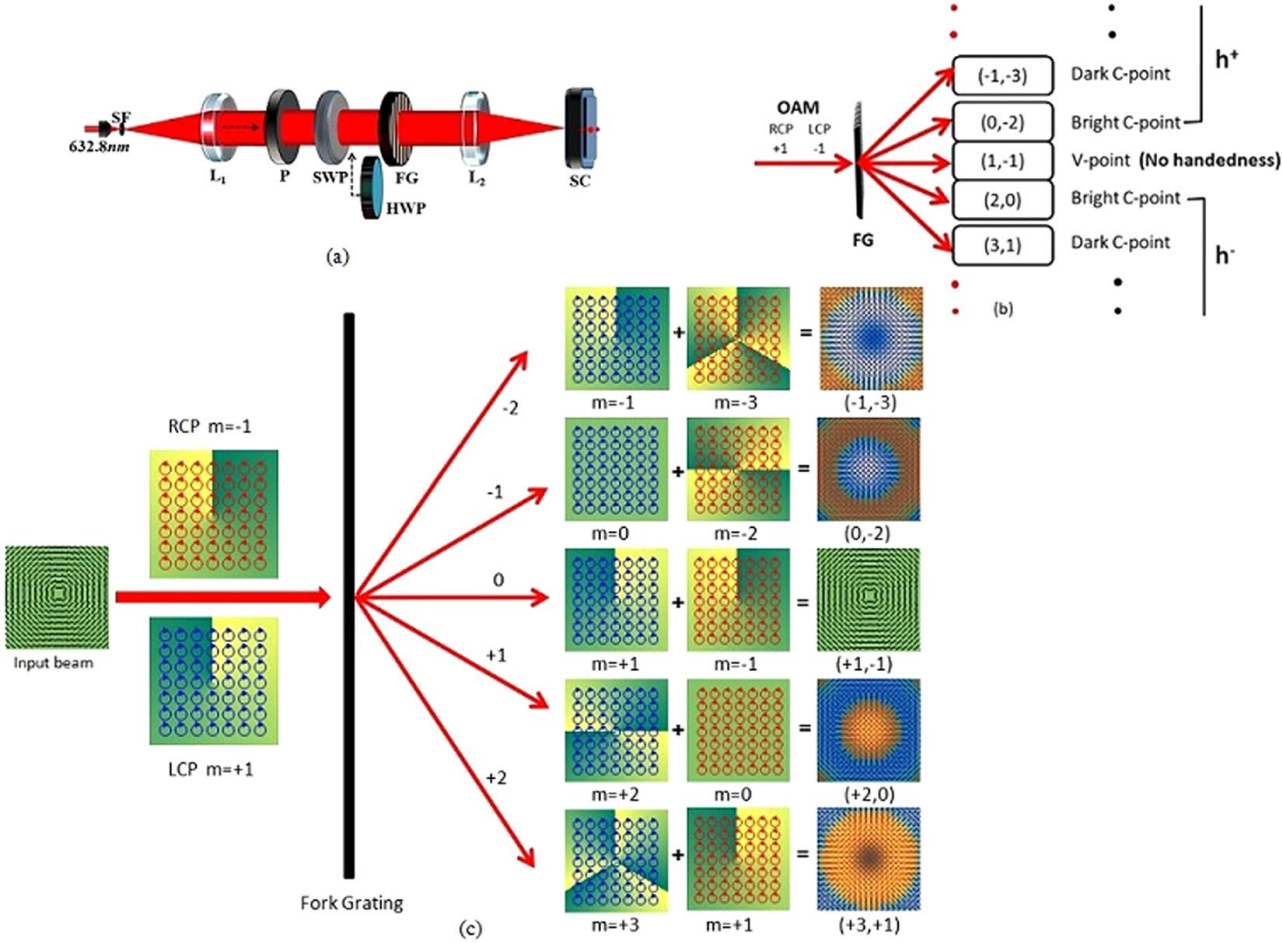
(**a**) Experimental Setup showing inhomogeneous SOP illumination of fork grating(FG) using S-waveplate (SWP), *L*_1_, *L*_2_: lenses, SF: Spatial filter, P: Polarizer and SC: Stokes Camera. (**b**) Schematic representation of the resultant OAM content and handedness in the different diffraction orders (**c**) Pictorial representation of the resultant OAM content and handedness in the different diffraction orders.

The V-point singularities with V-point index |*η*| = 1 can be classified into four types. The Jones vector notation and circular basis decomposition of these beams can be represented as^29^,

1 $${\hat{{\bf{J}}}}_{I}=[\begin{array}{l}-\sin (m\varphi )\\ \cos (m\varphi )\end{array}]=\frac{i}{2}\{{\psi }_{{m}^{+}}\,|{\sigma }^{-}\rangle -{\psi }_{{m}^{-}}\,|{\sigma }^{+}\rangle \}$$

2 $${\hat{{\bf{J}}}}_{II}=[\begin{array}{l}\sin (m\varphi )\\ \cos (m\varphi )\end{array}]=\frac{i}{2}\{{\psi }_{{m}^{+}}\,|{\sigma }^{+}\rangle -{\psi }_{{m}^{-}}\,|{\sigma }^{-}\rangle \}$$

3 $${\hat{{\bf{J}}}}_{III}=[\begin{array}{l}\cos (m\varphi )\\ \sin (m\varphi )\end{array}]=\frac{1}{2}\{{\psi }_{{m}^{+}}\,|{\sigma }^{-}\rangle +{\psi }_{{m}^{-}}\,|{\sigma }^{+}\rangle \}$$

4 $${\hat{{\bf{J}}}}_{IV}=[\begin{array}{l}\cos (m\varphi )\\ -\sin (m\varphi )\end{array}]=\frac{1}{2}\{{\psi }_{{m}^{+}}\,|{\sigma }^{+}\rangle +{\psi }_{{m}^{-}}\,|{\sigma }^{-}\rangle \}$$

where, ${\psi }_{{m}^{\pm }}$ = *e*^(±*imϕ*)^ are OAM modes with charge ±*m* and $|{\sigma }^{\pm }\rangle$ = [1 ± i]^*T*^ represents the Jones vector of SAM states for right/left circular polarization and T denotes the transpose. Here, types I and III are known as azimuthal and radially polarized beams (with *η* = 1) respectively. Types II and IV are their corresponding anti-vector modes (*η* = −1). A *q*^*th*^ order fork phase grating is generally obtained by realising a phase variation proportional to $\{(\frac{2\pi x}{{P}_{0}}+q\varphi )\}$, where the phase variations $\{\frac{2\pi x}{{P}_{0}}\}$ is that of ordinary 1D grating and {*qϕ*} is that of vortex of charge *q*. Using a phase SLM a fork phase grating whose transmittance function is *ψ*_*G*_ = $\exp \{ia(\frac{2\pi x}{{P}_{0}}+q\varphi )\}$, can be realised, where *a* decides the phase modulation. This fork grating (FG) produces optical vortex of charge (±*nq*) in the ±*n*^*th*^ diffraction order. Such a grating had been used to detect phase vortices in scalar optics^30,31^. Usually, when these gratings are illuminated with plane wave they will produce bright spot in the zero (0^*th*^) order at the focal plane. With vortex beam illumination, this bright spot accordingly shifts to ±*n*^*th*^ order. If the bright spot occurs at +*n*^*th*^ order then the charge of the incident beam is *m* = −*nq*. Now we consider the product of *ψ*_*G*_ with any of the V-point singularities given in Eqs (1–4), which generates helical wave *e*^(*i*(+*m*+*nq*)*ϕ*)^ and *e*^(*i*(−*m*+*nq*)*ϕ*)^ OAM states at *n*^*th*^ diffraction order in respective SAM states. V-points with PH index |*η*| = 1 will have two optical vortices of opposite charge (|*m*| = 1). Hence the diffracted beams will have the superposition of two optical vortices of charge (+*m* + *nq*) and (−*m* + *nq*) in *n*^*th*^ diffraction order. For the charge 1 FG, at *n* = −2 to +2 orders, the OAM composition of the beams in right and left circular states will be (−1, −3), (0, −2), (1, −1), (2, 0) and (3, +1) respectively as shown in Fig. 3(b). At 0^*th*^ order, the OAM combination is same as that of the incident beam. Hence, the superposition fields at respective diffraction orders can be written as,

5 $${\hat{{\bf{F}}}}_{-2}=\frac{A}{2}\{{\psi }_{{1}^{-}}\,|{\sigma }^{-}\rangle -{e}^{\{i{\alpha }_{-2}\}}{\psi }_{{3}^{-}}\,|{\sigma }^{+}\rangle \}$$

6 $${\hat{{\bf{F}}}}_{-1}=\frac{A}{2}\{{\psi }_{0}\,|{\sigma }^{-}\rangle -{e}^{\{i{\alpha }_{-1}\}}{\psi }_{{2}^{-}}\,|{\sigma }^{+}\rangle \}$$

7 $${\hat{{\bf{F}}}}_{0}=\frac{A}{2}\{{\psi }_{{1}^{+}}\,|{\sigma }^{-}\rangle -{e}^{\{i{\alpha }_{0}\}}{\psi }_{{1}^{-}}\,|{\sigma }^{+}\rangle \}$$

8 $${\hat{{\bf{F}}}}_{+1}=\frac{A}{2}\{{\psi }_{{2}^{+}}\,|{\sigma }^{-}\rangle -{e}^{\{i{\alpha }_{+1}\}}{\psi }_{0}\,|{\sigma }^{+}\rangle \}$$

9 $${\hat{{\bf{F}}}}_{+2}=\frac{A}{2}\{{\psi }_{{3}^{+}}\,|{\sigma }^{-}\rangle -{e}^{\{i{\alpha }_{+2}\}}{\psi }_{{1}^{+}}\,|{\sigma }^{+}\rangle \}$$

where, A = i for azimuthal and its anti-vector mode (Eqs 1 and 2) and A = 1 for radial and its anti-vector mode (Eqs 3 and 4). A half wave plate (HWP) can be used to get their anti-vector modes by switching the SAM states^16^. Here *α*_*n*_,(*n* = −2 to +2) is the relative Gouy phase delay between the SAM states for respective diffracted beams. Each vector beam will produce different combinations of such OAM and SAM states in various diffraction orders. In the non-zero diffraction orders, if the OAM content in both the SAM states are non-zero, dark C-point results.

To understand the difference between the beams observed at each diffraction orders, directly measurable Stokes parameters (*S*_0_, *S*_1_, *S*_2_, *S*_3_) are used. The Stokes field *S*_12_ = *S*_1_ + *iS*_2_ = *A*_12_ exp{*i*Φ_12_}, describes the nature of polarization singularity. In the Stokes phase Φ_12_, the polarization singularities appear as phase singularities. The charge of the Stokes phase vortex is called Stokes index. The Stokes index, ${\sigma }_{12}=\frac{{{\rm{\Delta }}{\rm{\Phi }}}_{12}}{2\pi }$ is related to the vector and ellipse field singularity indices as *σ*_12_ = 2*η* and *σ*_12_ = 2*I*_*c*_, respectively^17^. When the incident beam has |*η*| = 1, the central(zero) order will have |*η*| = 1 and all the other diffracted orders will have |*I*_*c*_| = 1. All the diffracted orders have same value of *σ*_12_. But the handedness of the C-points on either side of the zero order will be *h*^+^ and *h*^−^.

## Experiment and Results

Schematic experimental set-up is shown in Fig. 3a. In the experiment, we use He-Ne laser source (unpolarized *λ* = 632.8 *nm*). The laser beam is spatially filtered and a truncated plane beam is made using a spatial filter and lens assembly. The plane polarized light emerging from polarizer P is sent through an S-waveplate (SWP) which is composed of combined segments of Half Wave Plate (HWP) sectors with different fast axis orientations making an angle (*ϕ*) with *x*-axis^32,33^. This angle varies in azimuth fashion such that 0 ≤ *ϕ* ≤ *π*. Infact, SWP is a *q*-plate of charge (1/2)^34^. SWP can be used to produce Type -I and Type-III (azimuthal and radial V-points) both having positive Poincare-Hopf index. A HWP can be inserted into the beam to invert the sign of the Poincare -Hopf index so that Type-II and Type -IV singularities can be excited. Therefore SWP in combination with HWP can be used to generate all four degenerate states of V-point singularities. SWP can also produce an optical vortex beam when illuminated by circularly polarized light^25,35,36^. The beam with V-point singularity, generated from SWP is then passed through the FG of order 1, which transfers different OAM states depending on the diffraction order. The diffracted orders are then captured by the Stoke’s Camera (SC) placed at the focal plane of lens *L*_2_. This commercially available programmed camera, in quick succession, captures series of multiple frames of the same field. The series of multiple frames are in component polarization states viz., horizontal, vertical, diagonal, anti-diagonal linear component states and left and right circular polarization component states. Using these data the camera software can compute and provide the Stokes parameter distributions. Using MATLAB code, we compute the Stokes phase from the mathematically constructed complex Stokes field *S*_12_ = *S*_1_ + *iS*_2_ = *A*_12_ exp{*i*Φ_12_} and the SOP distribution. This phase distribution is useful in identifying polarization singularities in the diffracted fields. In Fig. 3(b), the segregation of handedness based on OAM transfer by diffraction is schematically presented. Figure 3(c) depicts the segregation of polarization singularities by diffraction according to their handedness, pictorially. In this depiction the phase and polarization are superimposed.

**Figure 4:**
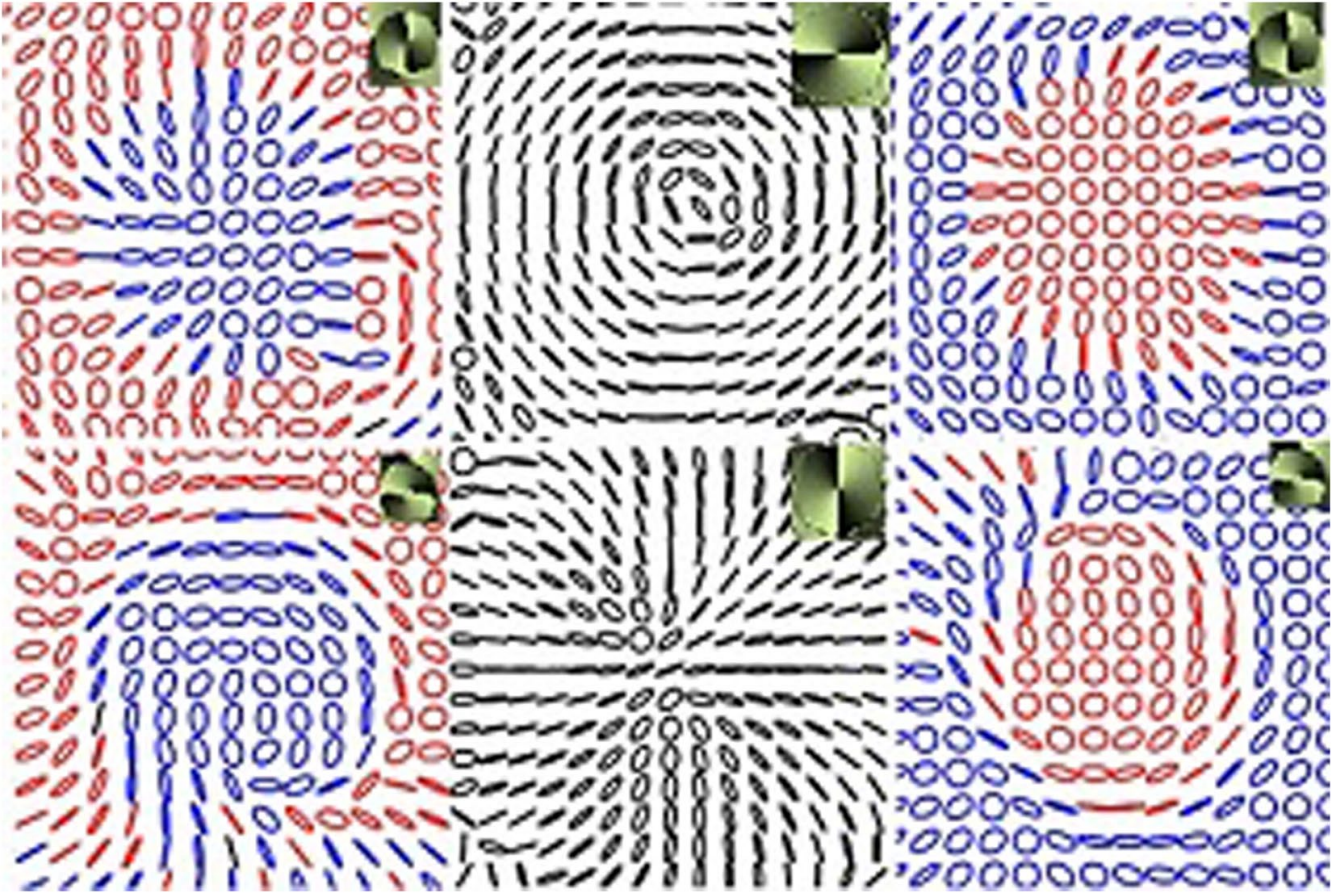
Experimentally observed SOP distributions in the −1,0, +1 diffraction orders from a fork grating illuminated by *η* = 1 beams. I row shows the results when the FG is illuminated by azimuthally polarized light. II row shows the results when the FG is illuminated by radially polarized light. Inset:Stokes phase.

**Figure 5:**
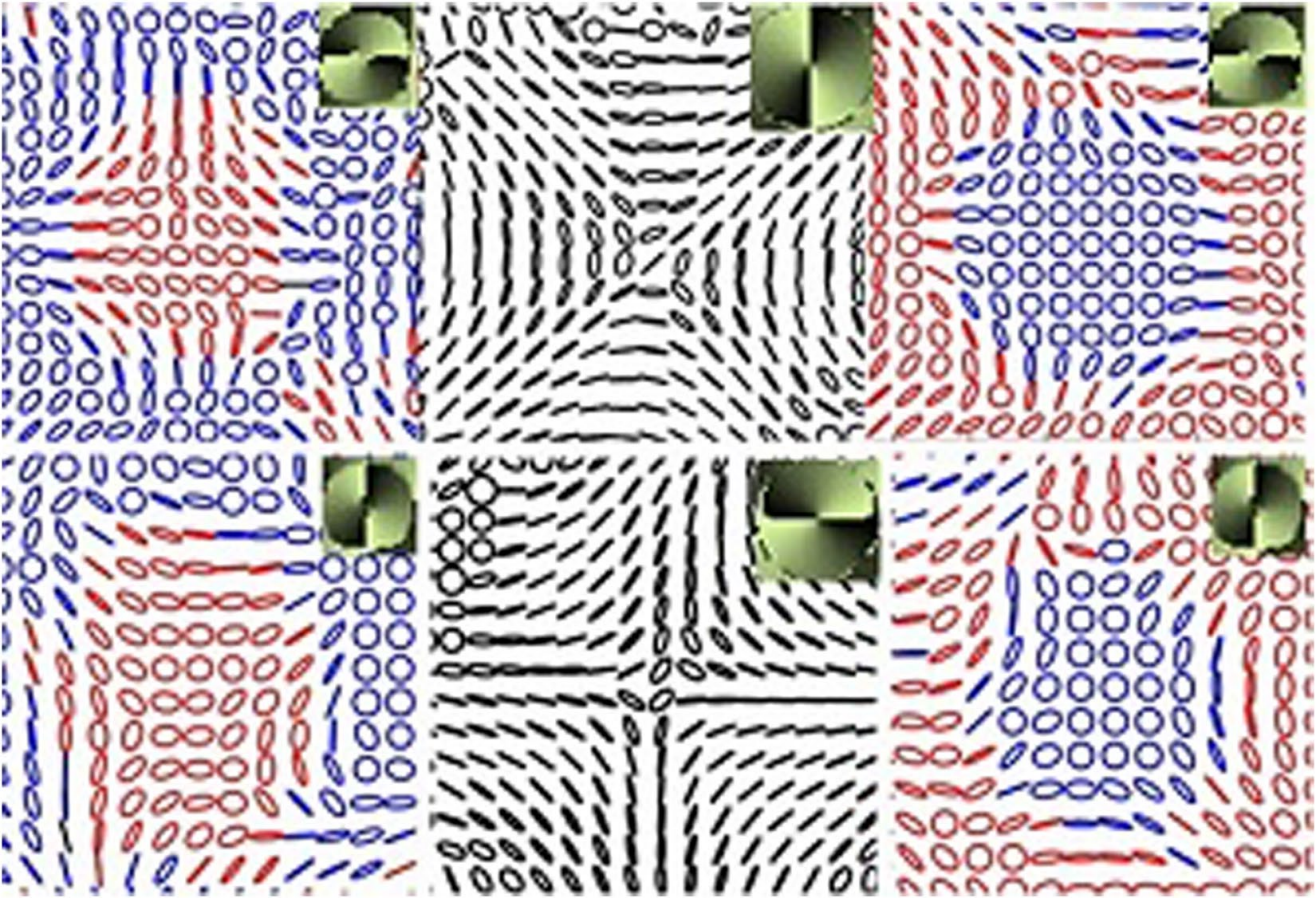
Same as Fig. 4 but the FG is illuminated with Type II (row 1) and Type IV (row 2) vector beams. Inset: Stokes phase.

Figure 4 depicts the experimental results of the radial and azimuthal beams diffracted through the fork grating. Stokes phase of each diffraction order is shown as inset. The diffraction intensity patterns look similar for all the four degenerate states of V-point singularities. Right handed and left handed polarization singularities are segregated into positive and negative orders. When the incident beam is an azimuthally polarized vector beam, all the diffracted orders are C-point singularities in which the major axes in their SOP distribution are radial. Similarly if the incident beam is a V-point singularity which is radially polarized then the major axes of the ellipses in SOP distributions of all the diffracted orders are azimuthally oriented. Negative PH index vector beams diffracted through fork grating show similar trend (i.e for Type II incident beam, the azimuth orientations in the diffracted beam are of Type IV and vice versa as shown in Fig. 5). The difference between the diffraction of positive and negative PH index vector beams is that the segregation of helicities into the positive and negative orders switch sides. It is surprising to note that the Stokes index *σ*_12_ is the same for all diffracted, undiffracted and incident beams even though there is OAM transfer.

## Discussion

Inhomogeneous polarization can host many types of singularities in the same beam. Singularities can be of finite size. So it is also possible to have both the handedness in the same beam separated by L-lines as shown in Figs 2, 4 and 5. These beams are similar to the Poincare beams where L lines separate regions of different handedness^37,38^. It can be seen in Figs 4 and 5 that the Stokes phase of the diffracted beams is rotated by *π*/2 with respect to the Stokes phase of the undiffracted beam. The incident V-point singularity and the diffracted C-point singularities are all superposition of different OAM states in opposite SAM states. Since the Gouy phase (=[|*m*| + 1]arctan(*z*/*z*_*R*_) is different for each OAM states^24^, the Gouy phase delay (GPD, *α*_*i*_ = |*m*_1_ − *m*_2_|arctan(*z*/*z*_*R*_) can be introduced between the two OAM states diffracted in a particular order. This GPD, in the present case is +*π* for positive, −*π* for negative and 0 for central diffraction orders. This additional phase (±*π*) due to GPD will be added to the Stokes phase of the undiffracted central beam, Φ_12_ = 2*ϕ* which in turn make *π*/2 rotation in the Stokes phase distribution affecting the polarization distribution across the beam^14,39^. Hence, when the incident polarization is radial/azimuthal (as is the same at central diffracted order), the polarization distribution across the off centered diffraction beams is azimuthal/radial. The first order beams possess non-zero intensity at the center. The other diffraction orders, due to difference in amplitude variation of the two superposing OAM beams, will have intensity nulls at the center making it to be a dark C-point^22^. It is interesting to note here that, even though the OAM composition of each beam is different all of them have same Stokes index *σ*_12_ = 2 which is true as *η* and *I*_*c*_ both are equal here.

**Figure 6:**
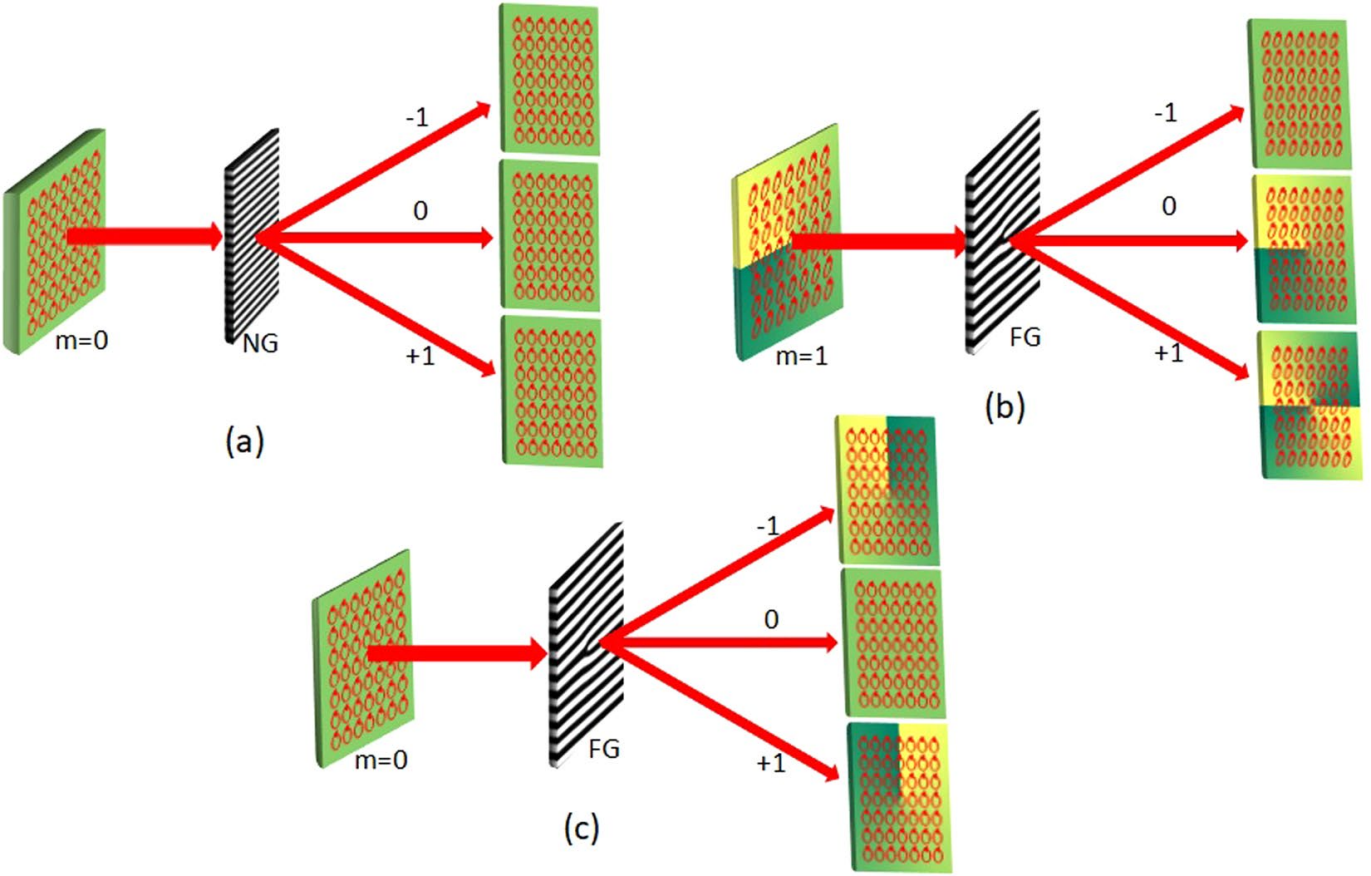
(**a**) Diffraction of circularly polarized beam through normal grating (**b**) Diffraction of elliptically polarized vortex beam through Fork grating and (**c**) Diffraction of circularly polarized beam through Fork grating.

To analyse how the helicity dependent diffraction happens we further carried out three more diffraction experiments to assert our observation. Let us consider the general case of diffraction of a homogeneously polarized (left/right circularly) beam through a normal grating. In this case, there is no OAM transfer involved. As expected all the diffraction orders have the same handedness and same polarization distribution as that of the incident beam which is shown in Fig. 6a. To understand whether just OAM involvement is enough to have helicity dependent diffraction, we considered two possibilities, one taking an incident beam having OAM and second, taking an incident beam not having OAM. To examine the former case we studied the diffraction of an elliptically polarized vortex beam through a Fork grating. Here even though the incident beam carries OAM due to the presence of vortex, the diffracted orders are still found to have the same handedness throughout as shown in Fig. 6b. For the latter case, a circularly polarized light beam was diffracted through a Fork grating. Here OAM arises when a homogeneous polarization distribution is diffracted by a Fork grating but still the positive and negative orders are observed to show the same handedness throughout as shown in Fig. 6c. These three experiments clearly indicate that just presence of OAM alone in definite spin states is not sufficient for helicity dependent diffraction. The important requirement is that the spatially varying polarization distribution should have OAM in the form of polarization singularity and this has to be transferred upon diffraction, which consequently leads to handedness segregation in the diffracted orders.

## Conclusion

In conclusion, we have experimentally shown for the first time that diffraction of a V-point singularity through a fork grating segregates the polarization singularities according to their handedness. The OAM transfer from the grating to the beam makes the diffraction of V-points helicity dependent. We also conducted three more experiments to confirm that this helicity dependent diffraction occurs only when the incident beam has a polarization singularity. These experiments are diffraction of (1) a circularly polarized beam through a normal grating, (2) an elliptically polarized vortex beam diffracted through a fork grating and (3) a circularly polarized light through a fork grating. In all these cases diffraction is not dependent on helicity. But when the incident beam has a polarization singularity, a Fork grating segregates the singularities as per their handedness. The Stokes index of the incident and diffracted beams are observed to be the same. Both simulation and experimental results are presented. We envisage the usefulness of our investigations on the helicity dependent diffraction in trapping and micro manipulations of the particles, segregation of chiral molecules^40^ and also for studying the light matter interaction, to mention a few.
