# Author Correction: Helicity dependent diffraction by angular momentum transfer

**DOI:** 10.1038/s41598-020-57949-0

**Published:** 2020-02-04

**Authors:** S. Deepa, B. S. Bhargava Ram, P. Senthilkumaran

**Affiliations:** Indian Institute of Technology Delhi, Department of Physics, New Delhi, 110016 India

Correction to: *Scientific Reports* 10.1038/s41598-019-48923-6, published online 28 August 2019

The version of this Article previously published contained lower resolution images for Figure 3. These have now been corrected in the PDF and HTML versions of the paper.

